# Dacryocystitis due to sporothrix inoculated vis an unusual mode: Case report

**DOI:** 10.1097/MD.0000000000011182

**Published:** 2018-06-22

**Authors:** Lixia Sun, Yaru Dong, Xin Wang, Baihui Shan, Min Zhang

**Affiliations:** a Department of Ophthalmology, Yanbian University Affiliated Hospital, Yanbian University, Yanji; b Department of Ophthalmology, Second Hospital of Jilin University, Jilin University, Changchun; c Department of Ophthalmology, Jiin Guowen Hospital, Gongzhuling; d Department of Dermatology; e Department of Pathology, Second Hospital of Jilin University, Jilin University, Changchun, Jilin, P.R. China.

**Keywords:** contact lens, dacryocystitis, sporotrichosis

## Abstract

**Rationale:**

Sporotrichosis is the most common subcutaneous mycosis. It is caused by the dimorphic fungus Sporothrix schenckii. Ocular sporotrichosis is uncommon and has been rarely reported.

**Patient concerns:**

We describe a 34-year-old female who presented with a nodule increasing in size near the medial angle of the left eye. Originally, she was misdiagnosed with a dacryocyst space-occupying lesion, and the lesion was removed by surgery.

**Diagnoses:**

Findings of fungal structures in the histopathological examination contributed to the diagnosis of Sporothrix dacryocystitis. Further culture of conjunctival secretions and contact lens storage solution was positive for *Sporothrix*.

**Interventions:**

She was treated with oral itraconazole, 200 mg by mouth twice daily.

**Outcomes:**

After 3 months of treatment with oral itraconazole, culture of the conjunctival secretions was negative.

**Lessons:**

It is of paramount importance to clinically suspect mycosis, even in unusual locations or in the absence of the typical epidemiological history.

## 1 Introduction

Sporotrichosis is a subacute or chronic mycosis, caused by traumatic inoculation of the *Sporothrix schenckii* fungus in most cases. It is the most prevalent subcutaneous mycosis in Latin America, with worldwide distribution.^[1]^ Infection is usually associated with traumatic subcutaneous inoculation with contaminated soil, plants, or organic matter contaminated with *Sporothrix*. *Sporothrix* mainly affects the exposed skin; the most common location is the upper limbs followed by the face, lower limbs, and chest.^[2]^ Ocular sporotrichosis is a rare condition, and even rarer cases of dacryocystitis caused by *Sporothrix* have been reported. In this article, we describe a case of *Sporothrix dacryocystitis* with the secondary involvement of both conjunctivae, probably due to auto-contamination with contact lenses (CLs).

## 2 Case history

**Figure 1:**
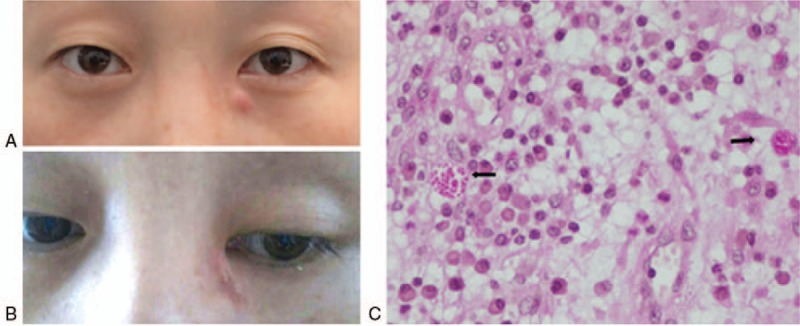
Facial photos of the patient and pathology result of the specimen, demonstrating (A) patient before and (B) 3 months after the surgery. A patient consent form has been obtained and archived; (C) Periodic acid-Schiff (PAS) staining of the specimen showed round fungal structures within the inflamatory infiltrate (400×).

**Figure 2:**
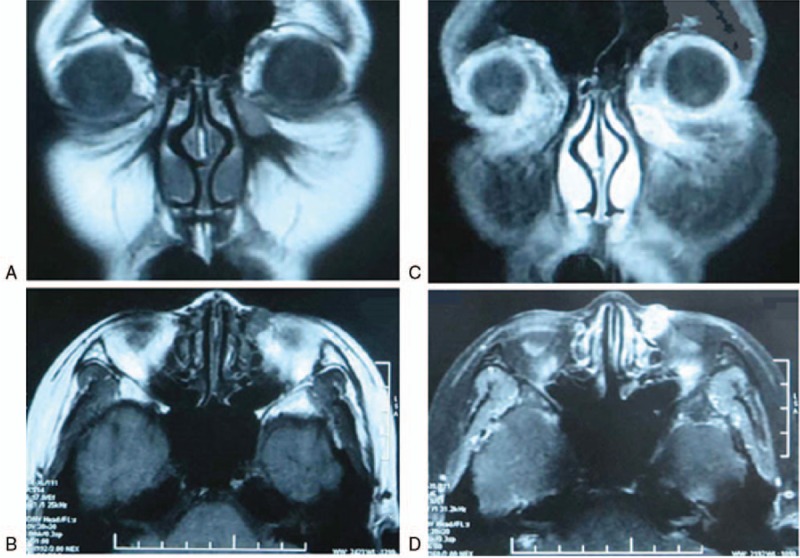
Magnetic resonance imaging (MRI) scan, demonstrating (A) Axial and (B) coronal sections of MRI scan and (C), (D) enhancement showing a tumor (10 mm×12 mm) with nonuniform density in the area of left dacryocystitis.

**Figure 3:**
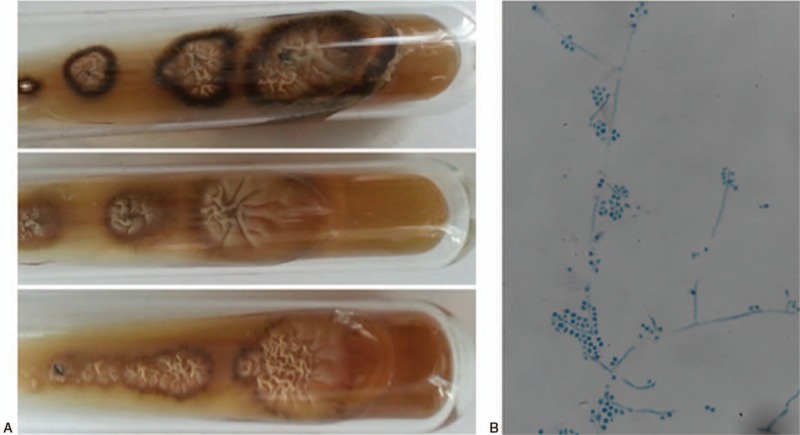
Fungal culture of specimens and microscopic examination of fungal cultures, demonstrating (A) fungal culture of specimens from the dacryocyst, conjunctival secretions and contact lens store solution at 3 d after inoculation, the Sabouraud Dextrose Agar showed whitish colonis with membranous aspect and surrounded by a blackened halo; (B) direct microscopic examination of fungal cultures showed branching, hyaline septate hyphae with oval conidia produced along the hyphae or in groups on short stalks. (400×).

A 34-year-old female presented to the ophthalmology department with a nodule near the medial angle of the left eye, progressively increasing in size for 1 month before presentation (Fig. 1A). She lived in an urban area in northeast China and denied contact with soil, plants, or animals, but she reported a history of wearing CLs for extended periods of time (more than 12 hours everyday for 2 years). The patient did not have any complaints of lacrimal discharge or epiphora. At the first examination, she had bilateral conjunctival injection and normal appearance of the puncta and canaliculi. The nodule was hard, without clear margins, and had poor mobility. Irrigation of the left lachrymal duct was unobstructed on the first day of hospitalization. Orbital computed tomography (CT) and magnetic resonance imaging (MRI) scans showed a space-occupying lesion (10 × 12 mm) in the left lacrimal sac area with nonuniform density (Fig. 2A, B) and non-homogeneous enhancement after contrast administration (Fig. 2C, D). Ultrasonography and X-ray examination of the main organs and systemic blood tests were normal. She was otherwise healthy. We suspected a dacryocyst space-occupying lesion and scheduled the patient for surgical excision of the lesion. Immediately before the operation, the left lacrimal excretory system was irrigated and found to be obstructed. Intraoperatively, we observed that the lesion tightly encapsulated the lacrimal sac, and the lesion along with the lacrimal sac was removed in their entirety and sent for pathologic examination. Histopathology demonstrated a diffuse, granulomatous, chronic inflammatory infiltrate in and around the lacrimal sac. Periodic acid-Schiff (PAS) staining of the specimen revealed rounded fungal structures within the inflammatory infiltrate, which were characteristic for spores from a fungal yeast (Fig. 1C). Culture of the specimen on Sabouraud Dextrose Agar at room temperature grew whitish colonies with membranous aspects surrounded by a blackened halo, identified as *S schenckii* after 3 days of incubation (Fig. 3A). Microscopy revealed branched, hyaline septate hyphae and daisy-like arrangements of conidia, showing characteristic features of Sporothrix (Fig. 3B). Further culture of the conjunctival secretions and CL storage solution was conducted in consideration of her binocular conjunctival injection, and both of them were positive for Sporothrix (Fig. 3A). On the basis of these findings, we modified the diagnosis to sporotrichosis. After the surgical procedure, she was treated with oral itraconazole, 200 mg by mouth twice daily. At a follow-up visit 3 months after the surgery, she remained asymptomatic, and a culture of her conjunctival secretions was negative (Fig. 1B); therefore, oral itraconazole was discontinued.

## 3 Discussion

Sporotrichosis is a chronic infection of the cutaneous and subcutaneous tissues and lymphatics caused by the thermally dimorphic subcutaneous fungus, *S schenckii*. Sporotrichosis has been traditionally known as “gardeners’ disease,” especially affecting persons who deal with soil, particularly in rural areas.^[3]^ There have been reports of mycosis following bites or scratches by animals, such as cats and squirrels, insect bites, and other injuries.^[4]^ Our patient was from Northeast China, an area of high epidemicity of sporotrichosis, but she lived in an urban area and denied contact with soil, plants, or animals. The disease mainly affects areas of the body exposed to trauma, such as the extremities and face.^[2]^ Ocular Sporotriosis infection may present various manifestations, which is associated with hematogenous dissemination or traumatic inoculation of contaminated soil, plants, or organic matter.^[5]^ Dacryocystitis is an unusual manifestation of sporotrichosis, which represents 0.18% of the sporotrichosis cases that were evaluated at the Evandro Chagas Institute of Clinical Research (IPEC) from July 2008 to July 2010, and most of them are from areas prone to infection with sporotrichosis such as Brazil.^[6]^ Although the pathogenesis of dacryocystitis is not completely understood, it typically occurs in the setting of nasolacrimal duct obstruction, and as a consequence, obstruction of the lacrimal ducts develops; therefore, epiphora and discharge are the most common presentations of dacryocystitis.^[7]^ We speculate that our patient was treated at the initial stage of dacryocystitis; therefore, she did not present with epiphora or discharge, and the lacrimal excretory system was unobstructed until just before the surgical procedure. Although less reported, conjunctival sporotrichosis can be seen in some cases.^[7–9]^ Different from other reported cases of conjunctival sporotrichosis, our patient first presented with a nodule near the medial angle of the left eye and bilateral conjunctival injection. We considered that her bilateral conjunctivae might be contaminated through any one of the following transmission channels: the fungus was transmitted from the nodule to the bulbar conjunctivae during the CL handling, which in turn contaminated the lens solution; or the fungus in the dacryocyst travelled retrograde to the conjunctivae along the lacrimal duct, then contaminated the CLs and lens solution during the CL handling. We are inclined to believe the second transmission channel for Sporothrix was found in the lacrimal sac but not skin surface of the nodule.

The lesion's unusual location and the patient's uncommon epidemiological history caused our misdiagnosis and mistherapy, which ultimately led to the loss of her dacryocyst. The polymorphic appearance of sporotrichosis lesions makes the diagnosis difficult on clinical grounds alone. In this particular case, sporotrichosis was definitively diagnosed by detection of rarely observed fungal structures via histopathological examination. Therefore, even in unusual locations or without a common epidemiological history, clinical suspicion of fungal diseases is of the utmost importance. The patient provided written informed consent for her case details, photos, and other images to be used for this report.

## Author contributions

**Conceptualization:** Yaru Dong.

**Data curation:** Lixia Sun, Yaru Dong.

**Formal analysis:** Lixia Sun.

**Funding acquisition:** Lixia Sun, Xin Wang.

**Investigation:** Xin Wang, Min Zhang, Baihui Shan.

**Methodology:** Xin Wang, Min Zhang, Baihui Shan.

**Project administration:** Lixia Sun, Xin Wang, Min Zhang, Baihui Shan.

**Resources:** Lixia Sun, Baihui Shan.

**Visualization:** Yaru Dong.

**Writing – original draft:** Yaru Dong.
